# Mediating Role of Negative Affectivity in the Association Between Lifetime Trauma and Gastrointestinal Symptoms

**DOI:** 10.3390/healthcare13070755

**Published:** 2025-03-28

**Authors:** Boukje Y. S. Nass, Pauline Dibbets, C. Rob Markus

**Affiliations:** 1 Department of Neuropsychology and Psychopharmacology, Faculty of Psychology and Neuroscience, Maastricht University, P.O. Box 616, 6200 MD Maastricht, The Netherlands; 2 Dr. Rath Health Foundation, 6422 RG Heerlen, The Netherlands; 3 Clinical Psychological Science, Maastricht University, P.O. Box 616, 6200 MD Maastricht, The Netherlands

**Keywords:** inflammatory bowel disease, irritable bowel syndrome, lifetime trauma, depression, anxiety

## Abstract

**Background/Objectives:** It is increasingly recognized that traumatic life experiences render individuals more vulnerable to gastrointestinal (GI) symptoms and chronic bowel conditions like inflammatory bowel disease (IBD) and irritable bowel syndrome (IBS). In this study, we examined whether this effect is mediated by negative affectivity. **Methods**: A total of 281 participants recruited in the Netherlands, including 94 with IBD, 95 with IBS and 92 controls, were assessed for lifetime trauma, trait anxiety, depression, and GI (IBD/IBS) disease activity. **Results**: The results confirmed that negative affectivity fully mediated the association between trauma and GI symptomatology, with trauma and depression explaining 38–40% (IBD|IBS) of the variance in disease activity and trauma and anxiety explaining 31–33% (IBD|IBS) of the variance in disease activity. Upon correction for condition (patient/controls), the predictive capacity increased even further, with trauma and depression now accounting for 43–44% (IBD|IBS) and trauma and anxiety for 40% (IBD and IBS) of the GI symptom heterogeneity. **Conclusions**: The results are in line with studies linking trauma to negative affectivity and negative affectivity to a more aggressive GI disease course. More generally, they show that the somatic and affective consequences of trauma should not be considered in isolation but must be treated as a covariant whole.

## 1. Introduction

It is increasingly recognized that traumatic life experiences contribute to the development of gastrointestinal (GI) symptoms and the exacerbation of chronic bowel conditions, including inflammatory bowel disease (IBD) and irritable bowel syndrome (IBS) [1,2,3]. However, they may not necessarily do so directly. Exploring the possibility of a mediated relation, the present study investigates the catalyzing role of negative emotions. That is, using path analyses, we verify whether stressful life experiences render individuals more vulnerable to recurring episodes of anxiety and depression which, in turn, increase their susceptibility to GI pathologies. This way, we hope to shed light on the complex interplay between emotional and physical (GI) health as well as their mutual dependence on (interdependence with) former life experiences, potentially paving the way for more effective (IBD/IBS) disease management and a more targeted approach for trauma-related bowel symptoms.

In support of the first path of the postulated mediational chain, there is indeed ample evidence linking stress and trauma to negative affectivity. Numerous studies have shown that trauma victims are more susceptible to mood and anxiety disorders [4,5,6,7]. Moreover, their proneness to feelings of anxiety and depression proved proportional to the number [7,8,9] and severity of the traumas they had endured, with the greatest negative affectivity being observed in the aftermath of domestic violence and physical/sexual abuse [10,11,12]. Additionally, trauma has been associated with poorer recovery and greater chronicity of negative emotions [13,14,15,16,17,18]. All in all, earlier findings support the alleged affinity between trauma and negative affectivity, suggesting that traumatic life experiences (especially those of a prolonged, invasive, early-onset, and cumulative nature) increase individuals’ affective reactivity, presumably as a function of trauma severity, chronicity, and accumulation.

Besides linking trauma to negative affectivity, the literature suggests that there is a strong interconnectivity between affective and GI states [19]. To illustrate, previous studies have shown that up to 35–75% of IBD patients (35% of those with quiescent and 75% of those with active disease) and 50–90% of IBS patients suffer from comorbid anxiety and/or depression [20,21,22]. Moreover, temporal changes in IBD activity were found to parallel fluctuations in affect [23], such that feelings of anxiety/depression peak during times of active disease [21,24,25,26] and subside during remissions [24]. Relatedly, comorbid anxiety and depression have been associated with a more aggressive GI disease course, higher pain rates, a lower quality of life (QoL), and poorer treatment (immunosuppression) responsiveness [27,28], which further validates the strong congruence/covariability between GI and affective states.

To recap, the existing literature postulates that trauma is a driving force in the development of both GI and affective disorders whilst also signaling that negative affectivity serves as a catalyst in the expression of GI symptoms. And yet, the three factors have hardly been examined in conjunction, leaving the precise role of negative affectivity in trauma-related GI complaints largely unexplained. That is, most studies have either focused on the association between affective and GI states irrespective of their shared connectivity to/joint affinity with prior life experiences [29,30] or isolated the somatic (GI) and psychological (affective) effects of trauma without considering their coexistence, interactivity, or sequential relatedness. This knowledge gap likely stems from the longstanding division between medical and psychosocial disciplines. As a result, the precise influence of negative affectivity on trauma-related GI symptoms remains largely unexplored and poorly understood. And yet, exploratory findings suggest that negative emotions channel the GI consequences of trauma and stress. To illustrate, it has been documented that childhood trauma predisposes IBS patients to feelings of anxiety and depression, which then interact with gastrointestinal symptoms to create a vicious cycle of gradually progressing GI and affective symptoms [31].

To further explore such detrimental inter-dynamics between trauma, negative affectivity, and GI vulnerability and bridge the interdisciplinary gap, the present study considers the three variables in conjunction, whereby anxiety and depression are expected to decisively determine (mediate) the association between trauma and GI symptoms. To this end, we first map the affective traits of IBS and IBD patients (i.e., Crohn’s disease (CD) and ulcerative colitis (UC), respectively) and compare them to those of GI symptom-free controls, assuming that both patient groups show a greater predisposition to anxiety and depression than the controls. Using mediation modeling, we then explore the direct and indirect (anxiety/depression-mediated) effect of trauma on GI disease, whereby it is expected that negative affectivity decisively determines (mediates) the association between trauma and GI (IBD/IBS) symptomatology. Lastly, subgroup analyses are conducted to determine whether the mediating influence of negative affectivity (anxiety/depression) is consistent across patient groups (IBS vs. IBD). I.e., even though one condition is organic and the other functional in nature, we hypothesize that trauma exerts its disease-aggravating effect through comparable (psycho-behavioral) pathways.

## 2. Materials and Methods

### 2.1. Study Sample

**Figure 1:**
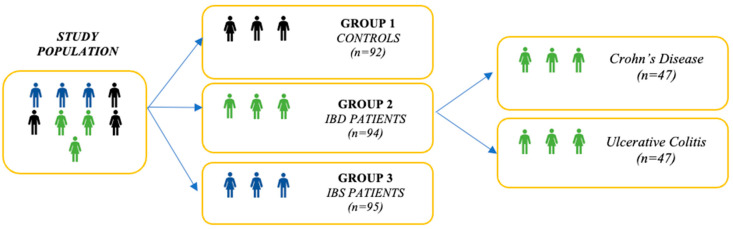
Overview of the study participants.

Since this study is part of a larger research initiative investigating mediators of trauma-related GI disturbances, all data were derived from an existing cohort in the Netherlands [32]. It included 189 GI patients (152 females, 37 males), 94 with IBD (Crohn’s disease: *n* = 47, 87.2% female, *M_age_* = 40.11, *SD_age_ =* 12.85; ulcerative colitis: *n* = 47; 66% female *M_age_ =* 47.19, *SD_age_ =* 12.25) and 95 with IBS (84.2% female, *M_age_ =* 43.06, *SD_age_ =* 14.47), complemented with 229 controls (81.2% female, *M_age_ =* 24.31, *SD_age_* = 11.67) (see Figure 1—a comparable figure has been published in [32]). Controls with significant GI symptoms (a clinically relevant score on one or multiple GI disease activity inventories) were excluded, reducing their numbers to 92 (whereof 71.7% female, *M_age_ =* 23.99, *SD_age_* = 11.40). Participants were enrolled through patient organizations, the Maastricht University medical center, social media, and the Maastricht University’s research participation system SONA. Only patients with a gastroenterologist-confirmed IBD or IBS diagnosis were eligible for inclusion in the patient groups.

### 2.2. Measures

#### 2.2.1. Negative Affectivity

Negative affectivity was assessed using the trait scale of the State–Trait Anxiety inventory and the Beck Depression Inventory (see more details below). While the former is the instrument of choice for measuring negative affectivity, it has been argued that the latter can serve as an inventory of negative affectivity as well, given the time frame used (two weeks) [33]. Together, the two instruments offer a more comprehensive assessment of participants’ emotional disposition, covering both dispositional anxiety and depression, two specific manifestations of the broader trait of negative affectivity.

The trait scale of the State–Trait Anxiety inventory (STAI-T). The STAI-T is a self-evaluation instrument composed of 20 statements monitoring an individual’s general tendency to experience anxiety and other negative emotions over time. It allows participants to indicate on a four-point scale how they generally feel (1—almost never to 4—almost always) [34,35], with total scores ranging from 20 to a maximum of 80. Here, a score of 44 serves as the previously validated cut-off, signaling dispositional anxiety [36]. It has been shown that trait anxiety scores covary with trauma [37,38] and GI symptoms [39,40], making the STAI-T an appropriate instrument for examining the mediating effect of trait anxiety on trauma-related GI symptoms. In the present study, the internal consistency of the STAI-T was satisfactory (α = 0.93).

The Beck Depression Inventory (BDI). The BDI is a 21-statement self-report measure, assessing the intensity of depressive symptoms experienced over the past two weeks [41,42]. Symptom manifestations are rated on 4-point Likert scales (0 = not present, 3 = frequent, severe), yielding a total depression score between 0 and 63 (with scores 0–9 marking normal ups and downs, 10–18 mild/moderate, 19–29 moderate/severe, and 30–63 severe depression). Evidence suggests that BDI scores covary with past trauma [43] and GI symptoms [44], making it a suitable instrument for studying the mediating effect of depression on the relationships between trauma and GI symptoms. In the present study, the internal consistency of the BDI was satisfactory (α = 0.89).

#### 2.2.2. Trauma Accumulation

Cumulative trauma exposure was assessed using the Dutch Life Events Questionnaire [45], a 28-item inventory mapping participants’ experiences with 12 potentially traumatic life events, namely divorce (parents/self), chronic illness, psychopathology, suicide attempts (parent/sibling/partner/child/self), death (parent/sibling/partner/child), domestic violence, substance abuse within the family, unwanted pregnancy, subjection to a crime, a serious accident, and sexual or physical abuse (self). Each item was administered trice, covering 3 separate life stages (<age 16, age 16 to one year ago, over the past year). Summed up, the items yield 3 life-stage-specific (range 0–28) and 1 lifetime score (range: 0–84).

#### 2.2.3. Clinical Measures of Disease Activity

Current GI disease activity was evaluated using three self-report inventories, two of which monitored IBD activity in the past week (UC and CD activity, respectively) and the third functional (i.e., IBS-related) GI symptoms over the past 10 days. Specifically, the IBD inventories encompassed the Patient Harvey–Bradshaw Index (P-HBI) [46] and the Patient-based Simple Clinical Colitis Activity Index (P-SCCAI) [47]. The P-HBI includes 11 items evaluating participants’ general wellbeing, abdominal pain, number of liquid stools, and extra-intestinal complications (arthralgia, uveitis, erythema nodosum, aphthous ulcer, pyoderma gangrenosum, anal fissure, new fistula, and abscess, respectively), with a total score ≥ 5 signaling active CD (5–7 mild; 8–16 moderate; and >16 severe disease). The P-SCCAI measures UC activity using 12 items that monitor bowel frequency, urgency of defecation, blood in stool, general wellbeing, and extra-intestinal manifestations, with a score of 3 or higher signaling active disease (score ≤ 2 = remission; >2 mild to moderate; >6 severe disease activity). Functional bowel symptoms were monitored using the Irritable Bowel Syndrome Symptom Severity Scale (IBS-SSS) [48], a 5-item (range 0–100) survey assessing abdominal pain (severity, days), abdominal distension, dissatisfaction with bowel habits, and quality of life, with a total score of 75 or higher signaling clinically relevant symptom presentations (75–175 indicating mild; 175–300 moderate; and >300 severe symptomatology).

### 2.3. Procedure

As this study is part of a broader research series on mediators of trauma-related GI disease activity, the study procedures are detailed in other publications [3,32]. In brief, individuals signed up via e-mail, whereupon they received a link to an online inventory package (Qualtrics) featuring the following assessment tools in chronological order: STAI-T, BDI, Dutch Life Events Questionnaire, Inadequacy scale, IBS-SSS, P-HBI, and P-SCCAI. This study was approved by the Ethics Review Committee Psychology and Neuroscience at Maastricht University (ERCPN-232_04_01_2021). Prior to study onset, all participants gave their informed consent.

### 2.4. Data Analysis

Data were first examined for missing values (SPSS version 28 for Mac). Next, *t*-tests were conducted to verify whether Crohn’s disease and colitis (CD/UC) patients differed in terms of their reporting of GI symptoms (P-SCCAI, P-HBI, IBS-SSS, BDI, STAI-T scores). Since no such group differences could be detected (*ts* < 1.55 *ps* > 0.12), Crohn’s and colitis patients were merged into a single IBD patient group for further analyses. Next, bivariate correlations revealed a large covariability between the two IBD activity index scores (P-SCCAI and P-HBI) (*r*(272) = 0.81, *p* < 0.001), allowing them to be pooled into a single Crohn–colitis activity composite score (P-HBI + P-SCCAI). By means of a one-way ANOVA, group differences (IBD|IBS|Controls) in negative affectivity (anxiety or depression scores) were examined. Multiple hierarchical regression analyses were conducted to explore the influence of negative affectivity (anxiety or depression) on the association between lifetime trauma and GI disease activity (pooled Crohn–colitis activity or functional bowel symptom severity). Here, total trauma (Model 1), negative affectivity (Model 2, anxiety or depression scores), and condition (dummy variable: 1 = patient, 0 = control) were sequentially added as predictors of disease activity (Crohn–colitis composite score or IBS-SSS score). Given the high correlation between anxiety and depression scores, they were only separately considered as predictors of disease activity. Significant regressions were further analyzed using Preacher and Hayes PROCESS mediation analyses (Model 4, bootstrapping with 5000 resamples). Since age correlated significantly with both outcome measures (Crohn–colitis composite score and IBS-SSS score) (*ps* < 0.001) and one mediator (BDI: *p* < 0.001), it was included as covariate in all analyses. Lastly, the multiple hierarchical regression and mediation analyses were repeated for subgroups separately (IBD patients/controls >< IBS patients/controls) to determine whether the influence of negative affectivity on the association between trauma and GI disease activity (pooled Crohn–colitis activity or functional bowel symptom severity) differed between patient groups.

## 3. Results

### 3.1. Sample Characteristics

Sample characteristics are presented in Table 1 (a comparable table has been published in [32]). At study entry, 50 percent of the CD patients showed active disease (P-HBI score > 5), 62 percent of the UC patients (P-SCCAI score > 2), and 95.8 percent of the IBS patients (IBS-SSS score > 74). Additionally, 70 percent of the CD and 80 percent of the UC patients reported functional GI symptoms (IBS-SSS score > 74).

### 3.2. Between-Group Comparisons

At study entry, more than 51% of the IBD patients showed a predisposition to depression (whereof 30% mild, 17% moderate, and over 4% severe) and 46.8% a predisposition to anxiety (STAI score 44 or higher). Of the IBS patients, more than two-thirds (68.4%) presented with dispositional depression (approximately 45% mild, 18% moderate, and 5% severe), and 58.9% presented with dispositional anxiety. As for the controls, 22.8% suffered from dispositional depression (mild symptoms only), and 33.7% suffered from dispositional anxiety. To further examine these group differences, a one-way ANOVA was performed, revealing a main effect of group (IBD|IBS|Controls) for depression (*F*(3, 277) = 15.84, *p* < 0.001) and anxiety (*F*(3, 277) = 7.82, *p* < 0.001) after correction for age. Pairwise comparisons using Bonferroni corrections confirmed that both patient groups suffered significantly more dispositional depression than the controls (*ps* < 0.001), with IBS patients also exhibiting greater trait anxiety (*p* < 0.001). Patient groups did not differ in terms of negative affectivity, *p*s > 0.08. As for lifetime trauma, observed group differences are described elsewhere [32]. In brief, both patient groups suffered significantly more cumulative trauma than the healthy controls, without differing from each other in that respect.

### 3.3. Cumulative Trauma and Negative Affectivity

As anticipated, cumulative trauma exposure was positively associated with both depression (patients: *r*(187) = 0.32, *p* ≤ 0.001; controls: *r*(90) = 0.46, *p* ≤ 0.001) and anxiety scores (patients: *r*(187) = 0.28, *p* ≤ 0.001; controls: *r*(90) = 0.24, *p* ≤ 0.05) among both patients and controls. That is, the more traumatized the individual, the greater his/her susceptibility to anxiety and depression.

### 3.4. Trauma and Depression as Predictors of GI Disease Activity

Multiple hierarchical regression analyses were conducted to examine the influence of depression on the association between cumulative trauma and GI symptom manifestations. The first hierarchical regression analyses, including total trauma (TT) as the sole predictor of changes in IBD activity (Model 1; Table 2), showed that trauma explained a significant proportion (17%) of the variance in disease activity (*F*(2, 271) = 28.084, *p* < 0.001; *R*^2^ = 0.172) after correction for age. When adding depression as an additional predictor (Model 2), the explanatory capacity of the regression more than doubled (*F*(3, 270) = 55.133, *p* < 0.001; *R*^2^ = 0.380), with TT no longer significantly predicting IBD activity. The addition of condition (dummy variable: patients = 1, controls = 0) as a third predictor (Model 3) yielded a further increase in explanatory capacity, with the regression model now explaining 43% of the variance in GI disease activity (*F*(4, 269) = 51.473, *p* < 0.001; *R*^2^ = 0.434).

**Figure 2:**
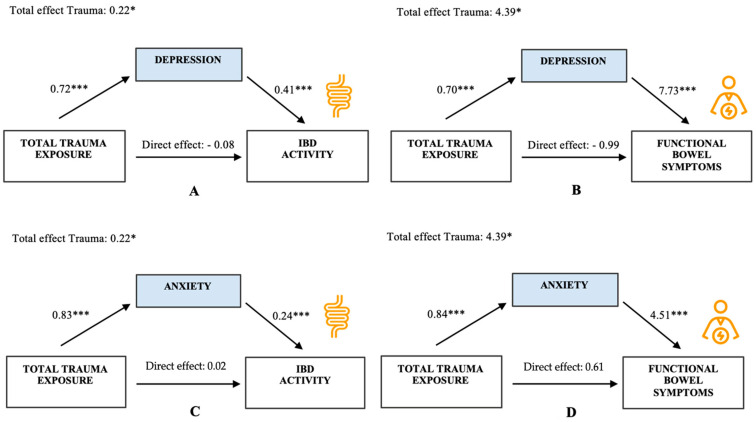
Mediation model. Direct and indirect effects of lifetime trauma on IBD activity (**A**,**C**) and functional bowel symptoms (**B**,**D**) through depression (**A**,**B**) or anxiety (**C**,**D**). All path estimates are unstandardized regression coefficients. * *p* < 0.05; *** *p* < 0.001.

To further examine significant regressions, PROCESS mediation analyses (Model 4) were conducted (Figure 2A), confirming that depression fully mediated the effect of cumulative trauma on participants’ (patients/controls) IBD activity (bootstrapped estimate = 0.29, 95% CI = 0.171, 0.417) when controlling for age. Hence, the greater the accumulation of trauma, the stronger individuals’ depression scores and, consequently, the greater their inflammatory disease activity. More so, upon correction for depression, the direct effect of TT on IBD activity ceased to be significant (*p* = 0.37), reaffirming the mere indirect influence of cumulative trauma on participants’ inflammatory disease activity.

Next, the same three-step hierarchical regression model was rerun to predict changes in functional bowel symptom severity (Table 3). The findings showed that total trauma (TT) as a sole predictor (Model 1) accounted for approximately 21% of the variance in functional bowel symptom scores after correction for age (*F*(2, 277) = 37.661; *p* < 0.001; *R*^2^ = 0.214). When adding depression as an additional predictor (Model 2), the explanatory capacity almost doubled, with TT and depression jointly accounting for 40% of the variance in functional GI symptoms (*F*(3, 276) = 60.085; *p* < 0.001, *R*^2^ = 0.395). When controlling for condition (dummy variable: patients = 1, controls = 0), both predictors explained as much as 44% of the inter-individual variance in functional bowel symptoms (Model 3: *F*(4, 275) = 53.416; *p* < 0.001, *R*^2^ = 0.437), suggesting that chronic bowel patients are particularly susceptible to the GI effects of trauma and depression.

PROCESS mediation analyses (Figure 2B) confirmed that depression fully mediated the association between TT and functional bowel symptoms (bootstrapped estimate = 5.38; 95% CI: 3.128, 7.676) after correction for age.

### 3.5. Trauma and Anxiety as Predictors of GI Disease Activity

Next, the influence of anxiety on the explanatory power of trauma in relation to IBD activity was examined. Hierarchical regression analysis including total trauma (TT) as the sole predictor of inflammatory bowel symptom presentations showed that trauma explained a significant proportion (17%) of the inter-individual differences in GI (IBD) disease activity (*F*(2, 271) = 28.084; *p* < 0.001; *R*^2^ = 0.172; Table 4) when controlling for age. When adding anxiety as a second predictor (Model 2), the model more powerfully explained 31% of the variation in disease activity, (*F*(3, 270) = 39.561; *p* < 0.001). The addition of condition as a third predictor (Model 3) resulted in a further 9% increase in explanatory capacity (*F*(4, 269) = 44.103; *p* < 0.001), with TT and anxiety now accounting for 40% of the variance in GI bowel symptoms, suggesting that chronic bowel patients are particularly sensitive to the GI effects of trauma and dispositional anxiety.

Subsequently, PROCESS mediation analyses corrected for age showed that the relation between TT and IBD activity was fully mediated by anxiety (bootstrapped estimate = 0.196; 95% CI: 0.107, 0.289) (Figure 2C).

Next, the same three-step hierarchical regression analysis was rerun to predict changes in functional bowel symptoms (Table 5). The results showed that total trauma (Model 1) accounted for 21 percent of the variance in functional bowel symptoms (*F*(2, 277) = 37.661; *p* < 0.001; *R*^2^ = 0.214) when correcting for age. Upon correction for a possible additional influence of anxiety (Model 2), the explanatory capacity of trauma increased significantly, with the regression model now accounting for 33 percent of the variance in functional bowel symptoms (Model 2: *F*(3, 276) = 45.067; *p* < 0.001, *R*^2^ = 0.329). When also controlling for condition (dummy variable: patients = 1, controls = 0), TT and anxiety explained up to 40 percent of the inter-individual variance in functional bowel symptoms (Model 3: *F*(4, 275) = 45.868; *p* < 0.001, *R*^2^ = 0.400).

Subsequently, the PROCESS mediation analysis revealed that the negative effect of trauma on participants’ functional bowel symptom severity was fully mediated by anxiety (bootstrapped estimate = 3.783, 95% CI: 2.088, 5.535) after correction for age (Figure 2D).

### 3.6. Additional Analyses

Lastly, all analyses were repeated to examine whether the mediating effect of negative affectivity differed between patient groups (IBS|IBD|Controls). As anticipated, the subgroup analyses yielded similar results. Yet, while total trauma (TT) and depression explained as much as 38 (IBD activity) to 43 percent (functional bowel symptoms) of the symptom variance in IBD patients (47–57 percent when controlling for condition), they explained up to 46 percent (functional bowel symptoms) of the symptom variance among IBS patients (79 percent when also considering the effect of condition). Similarly, TT and anxiety jointly explained 25 (IBD activity) to 32 (functional bowel symptoms) percent of the symptom variance in IBD patients (42–54 percent when also considering the concomitant effect of condition) while explaining no less than 41 percent (functional bowel symptoms) of the symptom variance among IBS patients (78 percent when controlling for condition). In other words, while the mediating function of negative affectivity was consistent across patient groups, the predictive power of trauma and negative affectivity proved to be greater for IBS patients.

## 4. Discussion

In the present study, the relationship between lifetime trauma, negative affectivity, and GI symptomatology was analyzed in a sample of chronic bowel (IBD|IBS) patients and GI symptom-free controls. Overall, the results confirm that negative affectivity fully mediates the relationship between cumulative trauma and GI disease.

First, we examined participants’ proneness to feelings of anxiety and depression, expecting that chronic bowel patients are more susceptible to recurring episodes of negative emotions than healthy controls. Confirmatively, up to half of all IBD patients showed predispositions for depression (51 percent) and anxiety (47 percent), as opposed to 23–34 percent of the controls. Among IBS patients, self-reported negative affectivity was even higher, with 68 percent exhibiting dispositional depression and 60 percent dispositional anxiety. These findings are consistent with previous research indicating that negative emotions are more common in IBD and IBS patients than controls [49,50,51]. Moreover, they affirm that the affective profiles of IBD and IBS patients are remarkably similar, despite the fundamental difference in their underlying pathophysiology (organic versus functional). That is, not only do both patient groups exhibit more cumulative trauma, maladaptive coping tendencies, and structural feelings of incapacity and uncontrollability than controls (as documented in [32]) but they are also more susceptible to feelings of anxiety and depression.

Next, we found that depression doubled the explanatory capacity of trauma in relation to GI disease activity. Specifically, lifetime trauma and depression jointly accounted for 38–40 (IBD|IBS scores) percent of the variance in GI symptoms (43–44 percent when the concurrent influence of the condition was considered). When examining IBD and IBS groups independently, the explained variance rose to 47–57 percent (IBD|IBS scores) for IBD patients and reached a substantial 79 percent (IBS scores) for IBS patients. Similarly, trait anxiety significantly increased the predictive power of lifetime trauma in relation to GI disease activity, with both factors jointly accounting for 31–33 percent (IBD|IBS scores) of the variance in GI disease activity (40–40 percent when the concurrent influence of condition is factored in). When analyzing the groups separately, the explained variance increased to 42–54 (IBD|IBS scores) percent for IBD patients and 78 percent (IBS scores) for IBS patients. In other words, while the mediating function of negative affectivity was consistent across patient groups, the predictive power of trauma and negative affectivity proved to be stronger for IBS patients. This likely stems from the distinct pathophysiology of functional versus organic bowel disorders. Functional symptoms primarily result from brain–gut axis disruptions, visceral hypersensitivity, and altered central pain processing, enabling a direct modulation of intestinal function by psychological factors [52,53]. Conversely, IBD’s strong organic, immune-mediated inflammatory nature, while aggravated by psychological and environmental influences, is not exclusively driven/governed by them [54,55].

Returning to the main hypothesis, we confirmed that structural changes in affect decisively determined (mediated) the association between lifetime trauma and GI disease activity. That is, the greater the accumulation of trauma, the greater the participants’ vulnerability to feelings of depression and anxiety and, consequently, the greater their (inflammatory and functional) GI disease activity. By implication, a meaningful effect of trauma on GI health is mainly to be expected in those in whom such trauma produces significant changes in affect (i.e., predisposes to recurring episodes of depression/anxiety). This observation is rather new. Yet, it aligns with the literature associating lifetime trauma to negative affectivity [10,11,12] and negative affectivity to a more aggressive GI disease course [56,57]. Additionally, it lends support to research indicating that negative affectivity serves as a mediator in the relationship between trauma and somatic symptoms [58]. Interestingly, while both anxiety and depression fully mediated the relationship between trauma and GI disease activity, depression emerged as the more significant risk factor. This aligns with findings suggesting that depression has a more significant effect on the physical morbidity of IBD patients compared to trait anxiety [59]. The underlying reason for this difference is likely to be found in a complex interplay of interrelated physiological and psychological processes that are more prominent in depression than anxiety. For example, from a biological perspective, depression shows stronger associations with various key physiological processes involved in the onset and exacerbation of GI disorders. These include reduced vagal tone [60,61], chronic activation of the hypothalamic–pituitary–adrenal axis [62,63,64], heightened pain perception through central sensitization [65], and systemic inflammation [66,67]. Synergistically, these processes exacerbate intestinal inflammation, increase gut permeability, alter motility, disrupt the gut microbiota, and enhance visceral hypersensitivity, worsening IBS and IBD symptoms accordingly. From a psychological standpoint, the core symptoms of depression, including profound sadness, emptiness, hopelessness, helplessness, and passivity, appear to have a more pronounced effect on GI health than the worry, fear, and dread typically associated with anxiety. This is supported by research indicating that IBD flares occur predominantly when patients experience feelings of helplessness, hopelessness, and powerlessness [68]. Furthermore, it is reinforced by the literature portraying GI symptoms as important somatic correlates (physical manifestations) of helplessness [69].

The present study is innovative as it considers the affective and somatic (GI) effects of trauma in tandem, presenting them as a covariant whole. Nonetheless, several limitations must be considered when interpreting the results. First, given the cross-sectional design of this study, caution is warranted when inferring causal relationships from the observed associations. I.e., even though our results are consistent with studies showing that traumas often precede changes in affect [8,9,10] and research suggesting that negative emotions have a stimulatory effect on GI dysfunctions [57], longitudinal studies are warranted to better comprehend the inter-dynamics between the three variables. Second, it is important to acknowledge the strong correlation between participants’ anxiety and depression scores, implying that both mediation pathways may have been operational. To verify whether participants develop GI health problems exclusively through one of the two mediation pathways, subgrouping analyses with larger samples are warranted. Relatedly, given the reciprocal relationship between GI and affective symptoms, it is highly probable that the causal pathways postulated here are in fact bilateral in nature. To validate this possibility, inverse mediation models were run, with lifetime trauma as the predictor, GI (IBD or IBS) disease activity as the mediator, negative affectivity (trait anxiety or depression) as the outcome measure, and age as the covariate. The findings affirm that trauma leaves people more vulnerable to GI distress and correspondingly to persistent feelings of anxiety and depression. Yet, while negative affectivity fully mediated the effect of trauma on GI (IBD/IBS) disease activity, the reverse pathway only showed partial mediation. Specifically, GI disease activity accounted for 19 (IBS)–18 (IBD) percent of trauma’s effect on depression and 17 (IBS)–18 (IBD) percent of its influence on anxiety. This asymmetry in mediation effects suggests that negative affectivity plays a more pronounced role in trauma–GI relationships compared to the reverse pathway. Nevertheless, the most likely scenario involves the activation of both pathways, whereby trauma serves as the catalyst for a self-perpetuating cycle of gradually progressing and mutually reinforcing GI/affective symptoms [31]. Third, this study relied on self-reported diagnostic and disease activity data only. As the COVID-19 pandemic restricted all possibilities for conducting in-person clinical and diagnostic assessments, it was not possible to complement these subjective disease activity indexes with objective biomarkers. Future research could strengthen our findings by incorporating biomarkers that enhance the reliability of our results. Fourth, subsequent studies may broaden their scope beyond negative affectivity assessments to include evaluations of adjustment disorder. By assessing both acute stress reactions (early indications of poor stress adaptation) and enduring psychological traits, we may be better able to predict long-term psychological, physiological, and immune-related health risks and develop targeted preventive strategies. Fifth, our sample was predominantly composed of female participants, potentially limiting the generalizability of our findings. However, it is worth noting that the female-to-male ratio was consistent across groups and aligned with broader population trends in these conditions [70,71,72]. Lastly, although our findings underscore the crucial role of negative affectivity, we cannot exclude that other factors influencing people’s stress reactivity also contribute to the observed effects. The intricate relationship between trauma and GI health likely involves multiple pathways and mechanisms, some of which may not have been captured in our current study. In future investigations, researchers should elaborate on other possible mediating factors. Additionally, upcoming studies would be enhanced by accounting for the use of psychotropic medications, as these can affect both psychological states and GI function.

## 5. Conclusions

All in all, the results suggest that chronic bowel patients are more susceptible to recurring feelings of anxiety and depression, which act as key mediators in the relationship between traumatic experiences and GI symptomatology. Our findings have several clinical implications. First, by outlining the affective vulnerabilities of chronic bowel (IBS| IBD) patients and relating them to inter-individual differences in GI (inflammatory/functional) disease activity, they offer new directions for (IBD/IBS) disease management. Second, by highlighting that these vulnerability factors (i.e., trait anxiety and depression) play a significant role in the relationship between lifetime trauma and GI disease, our findings provide new leads for managing the GI effects of trauma. Confirming this, preliminary studies have shown that behavioral and antidepressant therapies for affective and mood disorders have a concomitant positive effect on patients’ GI health and reduce their relapse risk [73,74]. Third, by revealing the strong interconnectivity between the somatic (GI) and affective consequences of trauma, they signal the need to consider and treat them concomitantly. Lastly, by showing how people’s former life experiences directly influence their (GI) disease susceptibility and morbidity later in life, they call for a broader perspective on health wherein the causes of disease or stalled recovery are not sought in the individual (or gastrointestinal tract) alone but rather in the individual-in-situation.

## Figures and Tables

**Table 1:** Sample characteristics.

		Group	
	IBD (*n* = 94)	IBS (*n* = 95)	Control (*n* = 92)
Female gender ^(%)^	76.6	84.2	71.7
Disease activity ^(M, Range)^			
CD patients (P-HBI score)	6.07 (0–20)		0.90 (0–4)
UC patients (P-SCCAI score)	4.51 (0–13)		0.67 (0–2)
Functional GI symptoms ^1 (M, Range)^	189.56 (0–441)	280.89 (50; 491)	22.49 (0–73)
Cumulative trauma ^(M, Range)^	5.51 (0–17)	6.47 (0–33)	3.43 (0–15)

^1^ = functional gastrointestinal symptom score as measured with the IBS-SSS, whereby higher scores indicate greater disease activity. P-HBI = Patient Harvey–Bradshaw Index, a CD activity index whereby higher scores indicate greater disease. P-SCCAI = Patient-based Simple Clinical Colitis Activity, a UC activity index whereby higher scores indicate greater disease. Crohn–colitis composite score = P-HBI score + P-SCCAI score.

**Table 2:** Hierarchical regression analyses testing the effects of lifetime trauma, depression, and condition on IBD activity (composite CD-UC activity scores).

	Step 1			Step 2			Step 3		
Predictor Variables	B	*t*	*β*	B	*t*	*β*	B	*t*	*β*
Trauma	0.215	2.320	0.140 *	−0.078	−0.901	−0.051	−0.033	−0.397	−0.021
Age	0.148	5.580	0.337 ***	0.125	5.405	0.285 ***	0.066	2.637	0.150 **
Depression				0.407	9.521	0.505 ***	0.358	8.515	0.445 ***
Condition							2.346	5.049	0.275 ***
*R*^2^ (Δ *R*^2^)	0.172 ***			0.380 (0.208) ***			0.434 (0.054) ***		
F Change	28.084 ***			90.651 ***			25.490 ***		

B, unstandardized regression coefficient; *t*, obtained *t*-value; *β*, standardized regression coefficient; *R*^2^, proportion of variance explained; Δ *R*^2^, change in proportion variance; * *p* ≤ 0.05; ** *p* ≤ 0.01; *** *p* ≤ 0.001.

**Table 3:** Hierarchical regression analyses testing effects of lifetime trauma, depression, and condition on functional GI symptom severity (FGISS).

	Step 1			Step 2			Step 3		
Predictor Variables	B	*t*	*β*	B	*t*	*β*	B	*t*	*β*
Trauma	4.394	2.423	0.142 *	−0.987	−0.580	−0.032	−0.324	−0.197	−0.010
Age	3.423	6.597	0.386 ***	3.028	6.609	0.341 ***	1.968	3.931	0.222 ***
Depression				7.725	9.095	0.469 ***	6.878	8.171	0.417 ***
Condition							42.264	4.539	0.244 ***
*R*^2^ (Δ *R*^2^)	0.214 ***			0.395 (0.181) ***			0.437 (0.042) ***		
F Change	37.661 ***			82.715 ***			20.604 ***		

B, unstandardized regression coefficient; *t*, obtained *t*-value; *β*, standardized regression coefficient; *R*^2^, proportion of variance explained; Δ *R*^2^, change in proportion variance; * *p* ≤ 0.05; *** *p* ≤ 0.001.

**Table 4:** Hierarchical regression analyses testing effects of lifetime trauma, anxiety, and condition on IBD activity (composite CD-UC activity scores).

	Step 1			Step 2			Step 3		
Predictor Variables	B	*t*	*β*	B	*t*	*β*	B	*t*	*β*
Trauma	0.215	2.320	0.140 *	0.019	0.217	0.013	0.045	0.537	0.029
	0.148	5.580	0.337 ***	0.160	6.532	0.363 ***	0.080	3.087	0.183 **
Anxiety				0.237	7.208	0.385 ***	0.221	7.162	0.358 ***
Condition							2.978	6.357	0.350 ***
*R*^2^ (Δ *R*^2^)	0.172 ***			0.305 (0.134) ***			0.396 (0.091) ***		
F Change	28.084 ***			51.955 ***			40.406 ***		

B, unstandardized regression coefficient; *t*, obtained *t*-value; *β*, standardized regression coefficient; *R*^2^, proportion of variance explained; Δ *R*^2^, change in proportion variance; * *p* ≤ 0.05; ** *p* ≤ 0.01; *** *p* ≤ 0.001.

**Table 5:** Hierarchical regression analyses testing effects of trauma (total), anxiety, and condition on functional GI symptom severity (*FGISS*).

	Step 1			Step 2			Step 3		
Predictor Variables	B	*t*	*β*	B	*t*	*β*	B	*t*	*β*
Trauma	4.394	2.423	0.142 *	0.611	0.346	0.020	0.954	0.570	0.031
Age	3.423	6.597	0.386 ***	3.674	7.627	0.414 ***	2.253	4.336	0.254 ***
Anxiety				4.505	6.877	0.358 ***	4.207	6.756	0.334 ***
Condition							53.812	5.721	0.311 ***
*R*^2^ (Δ *R*^2^)	0.214 ***			0.329 (0.115) ***			0.400 (0.071) ***		
F Change	37.661 ***			47.292 ***			32.729 ***		

B, unstandardized regression coefficient; *t*, obtained *t*-value; *β*, standardized regression coefficient; *R*^2^, proportion of variance explained; Δ *R*^2^, change in proportion variance; * *p* ≤ 0.05; *** *p* ≤ 0.001.

## Data Availability

The data presented in this study are available on request from the corresponding author.
